# Submicron-Bubble-Enhanced Focused Ultrasound for Blood–Brain Barrier Disruption and Improved CNS Drug Delivery

**DOI:** 10.1371/journal.pone.0096327

**Published:** 2014-05-02

**Authors:** Ching-Hsiang Fan, Hao-Li Liu, Chien-Yu Ting, Ya-Hsuan Lee, Chih-Ying Huang, Yan-Jung Ma, Kuo-Chen Wei, Tzu-Chen Yen, Chih-Kuang Yeh

**Affiliations:** 1 Department of Biomedical Engineering and Environmental Sciences, National Tsing Hua University, Hsinchu, Taiwan; 2 Department of Electrical Engineering, Chang-Gung University, Tao-Yuan, Taiwan; 3 Department of Neurosurgery, Chang Gung University College of Medicine and Memorial Hospital, Tao-Yuan, Taiwan; 4 Department of Nuclear Medicine, Chang Gung University College of Medicine and Memorial Hospital, Tao-Yuan, Taiwan; 5 Molecular Imaging Center, Chang Gung University College of Medicine and Memorial Hospital, Tao-Yuan, Taiwan; University of Hawaii, United States of America

## Abstract

The use of focused ultrasound (FUS) with microbubbles has been proven to induce transient blood–brain barrier opening (BBB-opening). However, FUS-induced inertial cavitation of microbubbles can also result in erythrocyte extravasations. Here we investigated whether induction of submicron bubbles to oscillate at their resonant frequency would reduce inertial cavitation during BBB-opening and thereby eliminate erythrocyte extravasations in a rat brain model. FUS was delivered with acoustic pressures of 0.1–4.5 MPa using either in-house manufactured submicron bubbles or standard SonoVue microbubbles. Wideband and subharmonic emissions from bubbles were used to quantify inertial and stable cavitation, respectively. Erythrocyte extravasations were evaluated by *in vivo* post-treatment magnetic resonance susceptibility-weighted imaging, and finally by histological confirmation. We found that excitation of submicron bubbles with resonant frequency-matched FUS (10 MHz) can greatly limit inertial cavitation while enhancing stable cavitation. The BBB-opening was mainly caused by stable cavitation, whereas the erythrocyte extravasation was closely correlated with inertial cavitation. Our technique allows extensive reduction of inertial cavitation to induce safe BBB-opening. Furthermore, the safety issue of BBB-opening was not compromised by prolonging FUS exposure time, and the local drug concentrations in the brain tissues were significantly improved to 60 times (BCNU; 18.6 µg versus 0.3 µg) by using chemotherapeutic agent-loaded submicron bubbles with FUS. This study provides important information towards the goal of successfully translating FUS brain drug delivery into clinical use.

## Introduction

The blood–brain barrier (BBB) presents a major obstacle to the entry of therapeutic molecules into the central nervous system (CNS) [1]. Recent experiments have confirmed that the permeability of the BBB can be increased by combining microbubbles with focused ultrasound (FUS) at frequencies ranging from 0.3 to 8.0 MHz [2]–[5]. The interaction between microbubbles and FUS temporarily deforms endothelial cells and changes the integration of tight junctions [6], [7]. This procedure leads to temporarily disruption of the BBB and thereby increases the efficiency of drug delivery locally in the CNS. This technology is ideally suited for the transcranial delivery of water-soluble drugs, and has the potential to deliver macromolecules (e.g. monoclonal antibodies) with up to tens of kilodaltons in size. Off-target effects are thus minimized in contrast to traditional systemic BBB-opening procedures such as osmotic BBB-opening through carotid infusion of a hypertonic solution [8], [9]. Furthermore, FUS-induced BBB opening is reversible within several hours, providing a window of opportunity for local delivery of therapeutic agents into the brain [10]–[15].

Despite the advantages of FUS-induced BBB opening, the interaction of microbubbles with FUS may occasionally be accompanied by side effects [4]. The most common side effect is brain tissue damage, which can be caused by ultrasound overexcitation (including excessive acoustic pressure, and/or duration of sonication) [16], or microbubble overdosing [17]. The presence of microbubbles in an ultrasonic field induces two modes of cavitation, stable and inertial. At low acoustic power levels, microbubbles oscillate within the ultrasound field and enlarge via rectified diffusion. Bubble activity of this type is called stable cavitation, and it induces stable bubble oscillation to emit acoustic harmonic signals (i.e. at harmonic or subharmonic frequencies) [18] and produce mild microstreaming forces in the bloodstream that stimulate surrounding endothelial cells [19]. Bubble activity with rapid growth and violent collapse under ultrasound exposure is referred to as inertial cavitation, which may be responsible for inducing erythrocyte extravasations, or possibly cell apoptosis, and tissue injury that accompanies FUS-induced BBB-opening [20], [21]. A number of studies have shown the importance of stable and inertial cavitation during BBB-opening process. For example, McDannold *et al.* have demonstrated that the threshold of BBB-opening was 0.29 MPa with ultrasound frequency of 0.26 MHz, whereas the inertial cavitation was induced exceeding 0.4 MPa [20]. Tung *et al.* have illustrated that the BBB-opening accompanied with harmonic activities which contributed to stable cavitation [21]. Liu *et al.* showed that erythrocyte extravasations occurred at high acoustic pressures (1.9 MPa) during the BBB-opening process [4], and erythrocyte extravasations further limited the permeability of BBB [10]. However, the exact roles of stable and inertial cavitation in BBB-opening and erythrocyte extravasations are not well confirmed, mainly because current micron-sized bubbles are polydispersed and can simultaneously undergo both kinds of cavitation activities under FUS exposure making it hard to characterize individual effects. The possibility of inducing erythrocyte extravasations is a major concern, and would greatly limit the application of repetitive microbubble-assisted FUS procedures aimed at increasing local drug concentration and delivery.

Therefore, the aim of this study is to propose a synergistic use of submicron bubbles (to greatly increase the threshold of inertial cavitation) and on-resonant frequency FUS (to amplify the stable cavitation activity) to maximize the separation of inertial and stable cavitation. Finally, the optimized FUS-enhanced chemotherapeutic agent delivery was performed by characterizing stable and inertial cavitation activities. Concurrence of BBB-opening and chemotherapeutic agents delivery for anti-glioma therapy has been verified by using multi-functional bubbles in our previous study [22]. Thus, another purpose of this study is to verify that a high concentration of chemotherapeutic agent (1,3-bis(2-chloroethyl)-1-nitrosourea, BCNU) can be safely and locally delivered by these submicron-sized drug-loaded bubbles upon FUS sonication. We compared the BBB-opening effect brought about by our in-house designed submicron bubbles to the traditional setup using commercial microbubbles. Cavitation activities were analyzed, and magnetic resonance imaging (MRI) and histological examinations were conducted to assess the occurrence of brain damages. Finally, the efficiency of drug delivery was determined by measuring the amounts of BCNU delivered into brain tissues by high performance liquid chromatography (HPLC).

## Materials and Methods

### Submicron Bubble Preparation

**Figure 1:**
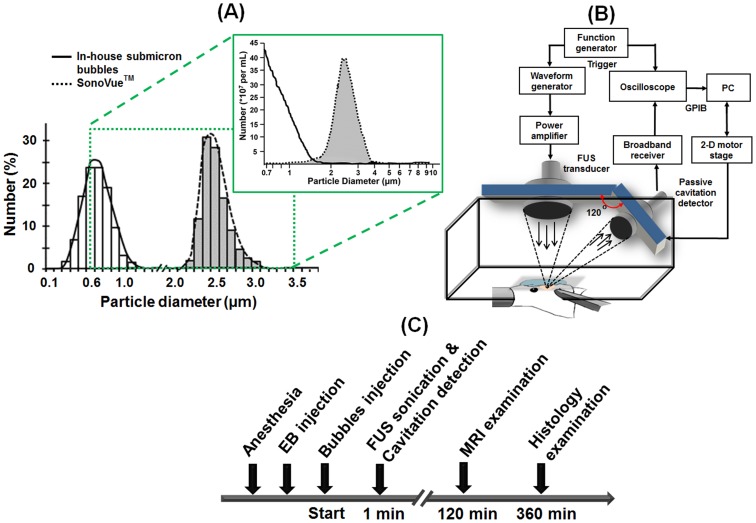
Experimental settings and procedures. A: Size distributions of in-house submicron bubbles and commercial SonoVue bubbles. Left diagram was measured by DLS and right one was determined by Coulter counter, which could provide both size distribution and concentration information. B: Experimental setup. C: Timeline of the *in vivo* experiment.

Submicron bubbles were prepared in-house via thin-film hydration method. Briefly, one milliliter of bubble formulation was prepared from 1,2-distearoyl-sn-glycero-3-phosphocholine (DSPC) (Avanti Polar Lipids, AL, USA) and 1,2-distearoyl-sn-glycero-3-phosphoethanolamine-N-[methoxy(polyethyleneglycol)-2000] (DSPE-PEG-2000) (Avanti Polar Lipids) at a molar ratio of 18∶1. The lipid mixture was dissolved in chloroform, which was then removed by evaporation in a rotary evaporator (Rotavapor R-210, Büchi Labortechnik AG, Flawil, Switzerland) so that a thin lipid film remained on the wall of the vial. The vial was lyophilized and stored at −20°C. To prepare the aqueous lipid solution, 1 mL of degassed 5 µL/mL glycerol in phosphate-buffered saline (PBS, pH 7.4) was added to the vial to dissolve the thin film. The lipids were dispersed by sonication for 5 min at room temperature. The sample was degassed in the same airtight vial which was then refilled with perfluoropropane gas (C_3_F_8_) and was heated to 65°C for 5 min. Submicron bubble suspensions were finally produced by intense shaking using a home-built agitator [23]. The bubbles were counted and sized using a Coulter counter equipped with a 30 µm sensor orifice (Multisizer 3, Beckman Coulter, Miami FL, USA) for a 0.7–20 µm range. Smaller particles (<1 µm) were measured by dynamic light scattering (DLS, Nanosizer-S, Malvern, London, UK). The mean bubble diameter was 1.10±0.07 µm and 0.65±0.01 µm by Coulter counter and DLS, respectively. The mean bubble concentration was (42.80±1.53)×10^9^ bubbles/mL by Coulter counter measurements (Figure 1A). The stability tests of submicron bubbles were listed in File S1 (Figure S1–S2). The resonance frequency of submicron bubbles were estimated at 10 MHz based on a standard procedure that literature has proposed (see Method S1, Table S1 and Figure S3–S4 in File S1) [24].

Commercially available microbubbles, SonoVue (Bracco Diagnostics Inc., Milan, Italy), had a mean diameter of 2.5 µm (resonance frequency of several megahertz) and were used at a concentration of approximately (3–5)×10^8^ bubbles/mL for comparison [25].

### Animal Preparation

All animal experiments were approved by the Institutional Animal Care and Use Committee of National Tsing Hua University and adhered to the experimental animal care guideline (IACUC approval number: NTHU10044). A total of 148 adult male Sprague-Dawley (SD) rats weighing about 300–350 g were studied. Animals were anesthetized intraperitoneally with chloral hydrate (400 mg/kg) before experiments. A cranial window of approximately 1×1 cm^2^ was fashioned with a high-speed drill before FUS irradiation to reduce distortion of the ultrasound beam. The skull defects were covered with saline-soaked gauze to prevent dehydration until the application of FUS. A catheter (PE50, Intramedic, Clay Adams Inc., NJ, USA) was inserted into the jugular vein to allow intravenous (IV) injection of bubbles, dyes and drugs. During the experiment, body temperature of the animals were maintained at 36±1°C by a heating pad (THM 100, Indus Instruments, Houston, TX, USA).

### Experimental Setup

The experimental arrangement is shown in Figure 1B. The FUS transducer and a passive cavitation detector (PCD) were arranged confocally using a self-made holder, with an included angle of approximately 120 degrees which was limited by their focal lengths and physical dimensions [21]. Two single-element FUS transducers with different center frequencies were used to transmit sonication pulses. The first FUS transducer was a single-element spherically-focused 10-MHz transducer (diameter: 25.4 mm, focus length: 52.2 mm, −6 dB bandwidth: 68.9%; V322, Panametrics, Waltham, MA, USA) and the second one was a single-element spherically-focused 1-MHz transducer (diameter: 25.4 mm, focus length: 52.7 mm, −6 dB bandwidth: 70.0%; V302, Panametrics). The PCD was a single-element spherically-focused 5-MHz transducer (diameter: 19.1 mm, focus length: 51.4 mm, −6 dB bandwidth: 57.1%; V308, Panametrics). The holder was mounted on a computer-controlled 2-D motion stage (HR8, Nanomotion, Yokneam, Israel). A waveform generator (model 2041, Tabor Electronics, Tel Hanan, Israel) was used to generate the transmitting pulse. A radio-frequency (RF) power amplifier (150A100B, AR, Souderton, PA, USA) amplified the pulses to drive the 1-MHz/10-MHz FUS transducers. The scattered echoes from bubbles were received by the PCD, and then amplified by a broadband receiver (BR-640, Retec, Warwick, RI, USA). The amplified RF signals were sampled at 25 MHz to accommodate the highest memory limit of the oscilloscope (LT354, LeCory Co., Chestnut Ridge, NY, USA) and then stored on the personal computer for off-line analysis. A function generator (33120A, Hewlett-Packard Co., Palo Alto, CA, USA) transmitted a trigger to synchronize the waveform generator used in sonication and the oscilloscope.

A water reservoir with a 4×4 cm^2^ opening at the bottom was used for animal experiments. The opening was sealed by a polyurethane membrane to allow the entry of ultrasound. The rats were laid prone under the water reservoir with their heads tightly attached to the membrane window. Ultrasound coupling gel (Aquasonic 100, Parker laboratories, Fairfield, NJ, USA) was applied between the cranial window and the membrane to maximize the transmission of ultrasound between the transducer and the brain. During the experiment, the animal was secured on the in-house stereotaxic apparatus with ear bars and a bite bar to reduce any undesired motion. The water in the reservoir was kept at 37°C by a heater to assist in maintaining the body temperature of the animals.

### FUS Calibration and Sonication

The acoustic pressure maps of the applied FUS transducers were measured in an acrylic water tank by using the FUS transducer attached to a semiautomatic 3-D positioning system. A polyvinylidene difluoride (PVDF) type hydrophone (HNP-0400, Onda, Sunnyvale, CA, USA; calibration range: 1–20 MHz) was used to measure axial and lateral acoustic pressure fields generated by the 10-MHz transducer in the water tank with which filled 25°C distilled/degassed water. The spatial resolution of the measurement was set to 300 µm. The measured half-maximum pressure amplitude of 10-MHz transducer at the focal zone had a diameter of 1.0 mm and length of 3.7 mm. The 1-MHz transducer was calibrated as the same way and the measured half-maximum pressure amplitude at the focal zone had a diameter of 2.9 mm and length of 24.8 mm. The acoustic pressure maps of 1-MHz, 5-MHz, and 10-MHz transduces were shown in File S1, Figure S5.

The resonance frequency of a gas bubble increases as its radius decreases [26]. To investigate the BBB-opening effect caused by different resonance frequencies of bubbles and excitation frequencies of FUS, we comprehensively perform verifications by testing all combinations of the submicron bubbles/SonoVue under the exposure of 1-MHz/10-MHz FUS energy to discriminate the playing roles of inertial cavitation/stable cavitation on BBB-opening/tissue damage. FUS sonication experiments were divided into four groups: (1) in-house submicron bubbles with 10-MHz FUS sonication; (2) SonoVue with 10-MHz FUS sonication; and (3) in-house submicron bubbles with 1-MHz FUS sonication; and (4) SonoVue with 1-MHz FUS sonication. Group 1 was used to verify that matching of the FUS frequency to the resonance frequency of the in-house submicron bubbles would maximize stable cavitation, Group 2 was used to verify that a large mismatch between the FUS frequency and the bubble resonance frequency resulted in no BBB-opening, Group 3 was used to demonstrate the BBB-opening caused by another off-resonant case of 1-MHz FUS excitation with the in-house submicron bubbles, and Group 4 was used to replicate the current common 1-MHz FUS protocol with SonoVue for comparison with Group 1. Detailed sonication conditions are summarized in Table 1. Pulsed-wave FUS was applied for 60 s by the 1-MHz/10-MHz transducer. For 10-MHz FUS sonication, acoustic pressure amplitudes of 0.5, 1.0, 1.5, 2.0, 2.5 and 4.5 MPa were investigated due to the importance of knowing the pressure threshold and minimizing inertial cavitation events. For 1-MHz FUS sonication, the rats were sonicated at 0.1, 0.2, 0.3, 0.5, 1.0 and 1.5 MPa. The cycle number of 1-MHz and 10-MHz FUS sonication experiments were set as a constant since we aimed to maintain the same oscillation number of bubbles during sonication. The center of the FUS focal zone was placed at 3.5 mm posterior and 2.5 mm lateral to the bregma, and 3 mm below the skull surface. The experimental protocol is shown in Figure 1C. A bolus of bubbles was injected 20 s prior to sonication. Each bolus injection contained a similar number of bubbles (1.5×10^7^) diluted to 0.2 mL with 0.9% normal saline solution for the two sets of bubbles. The bolus bubble injection was completed within 3 s. During sonication, the PCD setup was used to acquire acoustic-emission signals. About 120 min after sonication, rats were sent to the MRI scanning room to verify the occurrence of erythrocyte extravasations *in vivo*. After the experiments were completed, the brains were removed for histological examination.

**Table 1:** Sonication parameters of the four experimental groups.

	Group 1	Group 2	Group 3	Group 4
Number of rats	34	34	30	30
Acoustic pressure (MPa)	0.5–4.5	0.5–4.5	0.1–1.5	0.1–1.5
Pulse repetition frequency (Hz)	10	10	10	10
Number of cycles	10000	10000	10000	10000
Duration of sonication (s)	60	60	60	60
Number of MRI evaluations	18	18	18	18
Number of H&E staining evaluations	18	18	18	18
Number of TUNEL staining evaluations	18	18	18	18
Number of EB staining evaluations	34	34	30	30

Group 1  =  in-house submicron bubbles with 10-MHz FUS sonication; Group 2  =  SonoVue with 10-MHz FUS sonication; Group 3  =  in-house submicron bubbles with 1-MHz FUS sonication; Group 4  =  SonoVue with 1-MHz FUS sonication.

### Acquisition and Analysis of Passive Cavitation Detection (PCD)

**Figure 2:**
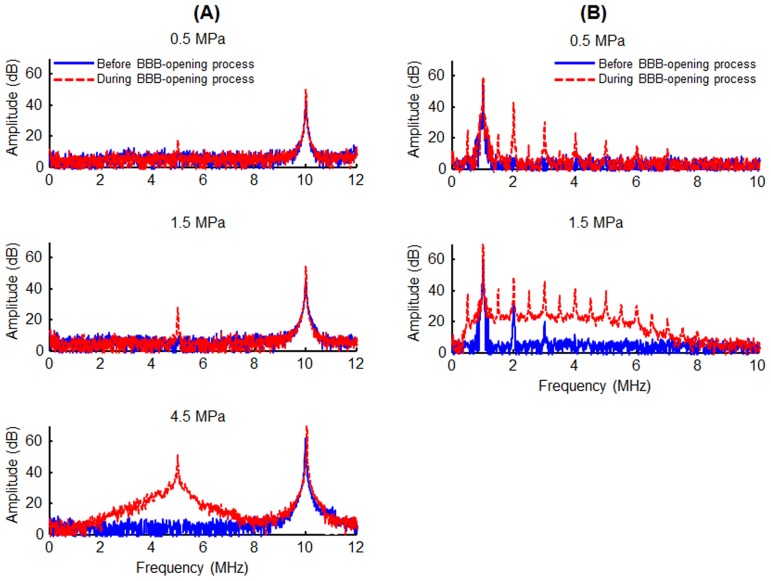
Acoustic-emission signal spectra. A: Frequency spectra acquired from PCD during 10-MHz FUS sonication with (red) or without (blue) in-house submicron bubbles. Top: 0.5 MPa, only the fundamental frequency (10 MHz). Middle: 1.5 MPa, the additional peak appeared at about subharmonic frequency (5 MHz). Bottom: 4.5 MPa, the spectrum was accompanied by wideband acoustic emission, indicated by a bump at approximately 2.2–6.8 MHz. B: Frequency spectra acquired from PCD during 1-MHz FUS sonication with (red) or without (blue) SonoVue. Top: 0.5 MPa. With injecting bubbles, both the fundamental frequency (1 MHz) and subharmonic signal (0.5 MHz) could be measured. Bottom: 1.5 MPa, with injecting bubbles, the frequency spectrum was accompanied by wideband acoustic emission, indicated by a bump at approximately 0.5–8 MHz.

The acoustic-emission signals collected by the PCD setup were used to quantify the subharmonic-frequency component and inertial cavitation dose (ICD) [27]. Note that the subharmonic-frequency component and ICD are indices of stable cavitation and inertial cavitation, respectively. In order to reduce the fragment of in-house submicron bubbles over time and bubble concentration decay during repeated ultrasound exposures, we primarily measured the acoustic-emission from the first excitation bursts. For each experimental group, the changes in the ratio of the acoustic-emission signals to the pressure amplitude were calculated. The acquired RF signals were analyzed to generate frequency spectra via fast Fourier transform with MATLAB (The Mathworks, Natick, MA, USA). The spectra consisted of energy from the scattered FUS signal (fundamental and higher-order harmonics; *n*×*f*, *n* = 1, 2, …, and *f* is the fundamental frequency), stable cavitation (sub- and super-harmonics frequency; *n*×*f*/2, *n* = 1, 2, …) and the radiated pressure from inertial bubble collapses (wideband frequency) [28]. The areas under the peaks of the subharmonic and wideband signals were measured to quantify stable and inertial cavitation dosages, respectively. For 10-MHz FUS sonication, the subharmonic signal area was estimated over the range of 4.85–5.15 MHz and the ICD was calculated as the area within the bandwidth of PCD (2.2–6.8 MHz), but excluded the subharmonic signal area (Figure 2A). For 1-MHz FUS sonication, the subharmonic signal area was estimated over the range of 0.35–0.65 MHz and the ICD was calculated within the area of 2.2–6.8 MHz but excluded the transducer's harmonic and ultraharmonic frequencies (Figure 2B). The change ratio in the acoustic emission signals (*AE*) was calculated by
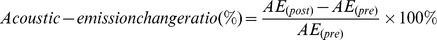
where *AE*_(*pre*)_ and *AE*_(*post*)_ are the areas of the spectrum with FUS exposure before and after bubble injection, respectively. The change ratios of acoustic-emission signals were also compared to the applied acoustic pressure amplitudes.

### Evaluation of BBB-opening

The permeability of the BBB was assessed by extravasation of Evans blue dye (EB; Sigma*-*Aldrich, St. Louis, MO, USA). This dye binds to albumin in the bloodstream, which prevents it from penetrating through the intact BBB. BBB-opening can be visualized as blue staining in brain tissues, and the level of BBB-opening can be estimated by quantitating the amount of EB extravasation into brain [17]. A bolus of EB (100 mg/kg) was injected 5 min prior to FUS sonication. Animals were sacrificed approximately 6 h after sonication and perfused with 0.9% normal saline via the left ventricle until colorless perfusion fluid appeared from the right atrium. Rat brains were then removed and sliced into coronal sections for observation. EB-stained brain samples were also withdrawn to measure the amounts of EB extravasation. The samples were weighed and then soaked in 50% trichloroacetic acid. After homogenization and centrifugation, the extracted dye was diluted with ethanol (1∶3) and the fluorescence was measured using a spectrophotometer (Cytofluor 2300, Millipore, Bedford, MA, USA; 620-nm excitation and 680-nm emission). EB extravasation in the brain was quantified by a linear regression standard curve derived from seven concentrations of the dye, and was expressed as nanounits per gram of tissue. All results are presented as mean and standard error of the mean values. The amount of EB extravasation was compared to the change ratio of acoustic emission signals. The BBB opening pressure threshold is the pressure level at which 100% sonicated animals underwent BBB permeability increase.

### MRI Examination

We used two different MRI protocols to verify the occurrence of brain damage *in vivo* by a 3-T MRI system (Trio with Tim, Siemens, Erlangen, Germany) and a standard wrist coil with an inner diameter of 13 cm. The first protocol was a T2-weighted sequence, which was used to confirm the absence of anatomical changes after sonication [4]. The following parameters were used: TR/TE = 3730 ms/115 ms, slice thickness = 1 mm, matrix size = 168×384, field of view (FOV) = 80×170 mm, which gave a voxel size of 0.4×0.4×1 mm^3^. The total acquisition time was 6 min and 3 s for six averages. The second protocol was a susceptibility-weighted imaging (SWI) sequence, which was used to determine the distribution of erythrocyte extravasations in the brain. The SWI sequence was modified from a heavy T2*-weighted gradient-recalled 3-D fast low-angle shot sequence with full flow compensation in all three directions. The SWI sequence was acquired with the following parameters: TR/TE/flip angle = 28 ms/20 ms/15°, slice thickness = 0.7 mm, number of slices = 16, matrix size = 256×384 and FOV = 80×130 mm, which gave a voxel size of 0.3×0.3×0.7 mm^3^. The acquisition time for single measurement was 2 min 24 s. Ten measurements of the SWI sequence were acquired and averaged to increase the signal-to-noise ratio. During MRI imaging process, animals were anesthetized with a mixture of oxygen (0.8 L min^−1^ at 1.0 Bar, 21°C) and 2% vaporized isoflurane using an anesthesia vaporizer.

### Brain Damage Evaluation

The removed brain was fixed in 10% neutrally buffered formalin (Sigma-Aldrich) followed by embedding in optimal cutting temperature compound (Tissue-Tek OCT, Sakura Finetek Inc., Torrance, CA, USA) and storage at −50°C. The brain samples were serially sectioned with a slice thickness of 15 µm. The direction of these slices was the same to FUS sonication direction. The bluest section was identified and subjected to hematoxylin and eosin (H&E) staining to confirm the presence of erythrocyte extravasations. In addition, terminal deoxynucleotidyl transferase biotin-dUTP nick end labeling (TUNEL, ApopTag kit, Intergen Co., Purchase, NY, USA) was used to detect apoptotic neurons [29]. Histology evaluations were performed using light microscopy by a person who was blinded to the ultrasound parameters but was informed of sonication location of the brain.

The extent of brain damage was rated according to a four-point scale as follows [3], [4]: grade 0 = no tissue damage or erythrocyte extravasation; grade 1 = presence of few extravasated erythrocytes, but no neuronal loss; grade 2 = presence of extensive erythrocyte extravasations; and grade 3 = extensive erythrocyte extravasations along with apoptotic neuronal death.

### Synthesis of Submicron-Sized BCNU-Loaded Bubbles

To synthesize BCNU-loaded bubbles (BCNU-bubbles), dipalmitoyl-phosphatidyl-choline (DPPC) (Avanti Polar Lipids) and DSPC-PEG-2000 at a molar ratio of 19∶1 were mixed with 37.6 µL BCNU solution (100 mg BCNU dissolved in 3 mL purified ethanol) (Bristol-Myers Squibb, NY, USA) in chloroform in a vial. The organic solvent was eliminated at 0–4°C under reduced pressure over 24 h using a rotary evaporator. The procedure for preparing BCNU-bubbles was almost the same as that used to prepare submicron bubbles. The mean diameter of BCNU-bubbles was approximate 0.86±0.18 µm by DLS and 1.16±0.11 µm by Coulter counter, and the concentration was (19.78±4.9)×10^9^ bubbles/mL by Coulter counter.

BCNU encapsulation efficiency was analyzed using a reverse method by HPLC with a UV detector (L-2400, Hitachi, Tokyo, Japan) [22]. BCNU-bubble formulations were gently centrifuged for 3 min at 6000 rpm (mini-micro centrifuge, Bertec Enterprise Co., Taipei, Taiwan). The underlying liquid phase was carefully separated from the top foam cake with a syringe needle and the residual BCNU after production of BCNU-bubbles (*W_res_*) was determined. Drug payload (*W_BCNU-B_*) was calculated as the difference between the total amount of BCNU (*W_tot_*) and *W_res_*. Drug encapsulation efficiency was then calculated by the equation:
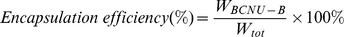



The mobile phase solution consisted of 60 vol% HPLC-grade methanol in deionized water. A column packed with mightysil RP-18 GP 250-4.6 (Kanto Chemicals, Tokyo, Japan) was used with a flow rate of 2.0 mL/min and a detection wavelength of 237 nm. The amount of BCNU was quantified by the area under its characteristic peak at a retention time of 3.2 min.

### Quantitative Determination of BCNU Accumulation in Brain Tissue

The feasibility of enhanced local BCNU delivery by BCNU-bubbles and repetitive sonication was investigated by delivery of 10-MHz FUS transcranially in the presence of BCNU-bubbles. Experiments with four different FUS exposure conditions were conducted: (1) 1.5-MPa exposure (60 s) with BCNU-bubbles; (2) 2.5-MPa exposure (60 s) with BCNU-bubbles; and (3) 2.5-MPa exposure (240 s) with BCNU-bubbles; and (4) 4.5-MPa exposure (240 s) with BCNU-bubbles. Besides, two experiments including BCNU administration-only (without FUS exposure) as well as BCNU-bubbles administration only (without FUS exposure) were also conducted to serve as the reference groups. The detailed sonication parameters are described in Table 2. Twenty-four skull-intact SD rats were used. Before sonication, the fur on the top of the head was completely removed using an electric trimmer. Rats were IV injected with 0.5 mL of BCNU-bubbles diluted to 1 mL with 0.9% normal saline. Sonication started 20 s after injection of BCNU-bubbles at the left hemisphere brain and the untargeted right hemisphere brain was used as a control.

**Table 2:** Summary of 10-MHz FUS with BCNU-bubbles for enhanced BCNU delivery.

	BCNU	BCNU-bubble w/o FUS	Case 1	Case 2	Case 3	Case 4
Number of rats	4	4	4	4	4	4
Acoustic pressure (MPa)	-	-	1.5	2.5	2.5	4.5
Pulse repetition frequency (Hz)	-	-	10	10	10	10
Number of cycles	-	-	10,000	10,000	10,000	10,000
Number of sonication sites	-	-	1	1	1	1
Duration of sonication per site (s)	-	-	60	60	240	240

### Quantitation of BCNU Accumulation in Brain Tissue

Animals were sacrificed after FUS exposure, and the concentration of local BCNU deposited in the brain hemisphere was measured. To minimize the loss of BCNU caused by hydrolysis *in vivo*, rat brains were perfused with saline and removed within 10 min after FUS sonication. Procedures were performed at 0–4°C to reduce BCNU degradation. Each brain sample was homogenized in a 15 mL centrifuge tube with 0.5 mL methanol, and BCNU was extracted in 2 mL methanol with a sonicator (model 2510, Branson Co., Danbury, CT, USA). After BCNU extraction, the samples were centrifuged at 13500 rpm for 10 min. The clear supernatants were collected and the precipitates were re-extracted twice. The final supernatants were filtered through a 0.2 mm filter before quantitative analysis and were finally analyzed by HPLC with UV detection. BCNU deposition was quantified using a linear regression standard curve derived from BCNU standard solutions (0.25–25 µg/mL).

### Statistical Analysis

Results are presented as the mean and standard error of the mean of at least three independent measurements. All statistical evaluations including the change ratio of acoustic-emission signals, the amount of EB extravasation, and BCNU uptake at the sonicated side of the brain were carried out with unpaired two-tailed Student's *t*-tests. A *p*-value of less than 0.05 (*p*<0.05) was considered significant. Statistical analysis was performed using Microsoft Excel 2010 (Microsoft, NY, USA).

## Results

### *In Vivo* Cavitation Detection

**Figure 3:**
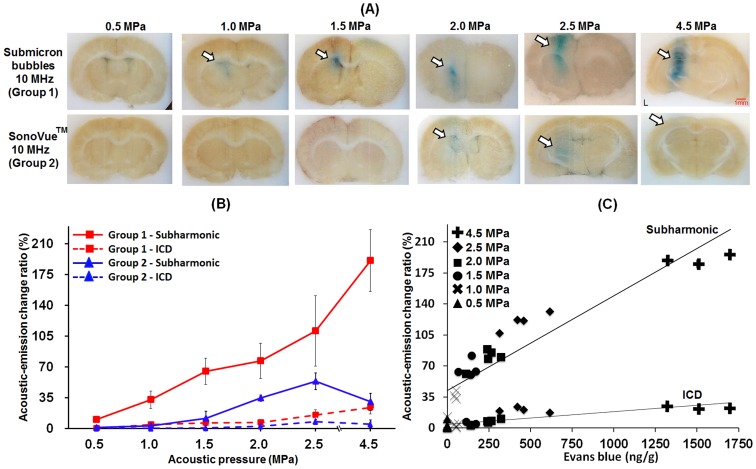
Relations between levels of BBB-opening and acoustic-emission signals under 10-MHz sonication. A: EB extravasation into brain tissue. (white arrows: the BBB-opening region; red arrows: areas of erythrocyte extravasations). B: Acoustic-emission change ratio (red line: in-house submicron bubbles with 10-MHz FUS sonication; blue line: SonoVue with 10-MHz FUS sonication; solid line: subharmonic-frequency signal; dotted line: ICD. C: Correlation between acoustic-emission signals and amount of EB extravasation for in-house submicron bubbles with 10-MHz FUS sonication.

In Group 1, BBB-opening was successfully induced with acoustic pressures above 1.0 MPa. No erythrocyte extravasations were observed in the gross histological sections. Stable cavitation activity increased profoundly when the acoustic pressure was increased to 1.0 MPa, whereas inertial cavitation activity remained reduced. By comparison, the results of Group 2 demonstrated that stable cavitation activity could be induced with mismatched the frequency of FUS, and therefore BBB-opening occurred at pressure of 2.0 MPa (Figure 3A and 3B). From the observation of brain section, the combined use of 10-MHz FUS and submicron bubbles has the most profound BBB-opening effect. However, SonoVue excited at 10-MHz with 4.5 MPa did not reproduce the BBB-opening. It should be noted that the PCD measurements indicated that the activity of stable cavitation increased significantly from 0.5 to 2.5 MPa, but decreased at 4.5 MPa (Figure 3B). The possible reason is that the micrometer-sized bubbles could not sustain under the high pressure and gas bubbles diffused quickly [30]. The gas-diffused bubbles could not vibrate and expand effectively, and thus did not induce enough cavitation dosage to stimulate surrounding vessels.

**Figure 4:**
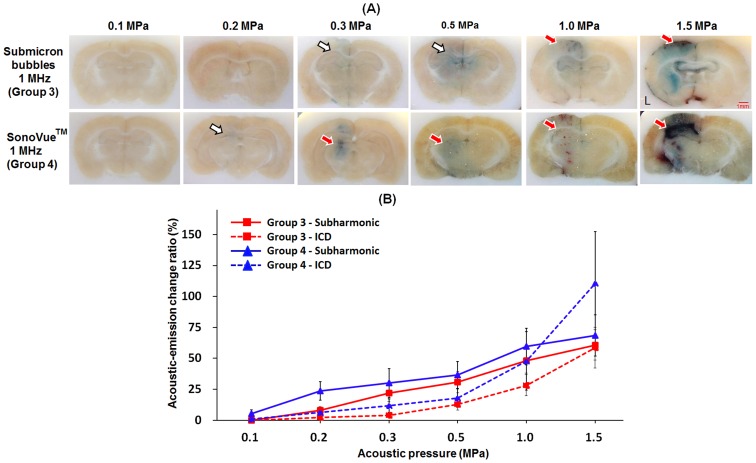
Relations between levels of BBB-opening and acoustic-emission signals under 1-MHz sonication. A: EB extravasation into brain tissue. (white arrows: the BBB-opening region; red arrows: areas of erythrocyte extravasations). B: Acoustic-emission change ratio (red line: in-house submicron bubbles with 1-MHz FUS sonication; blue line: SonoVue with 1-MHz FUS sonication; solid line: subharmonic-frequency signal; dotted line: ICD.

In Group 3, the occurrence of BBB-opening and stable cavitation started from at 0.3 MPa. Because inertial cavitation activity increased along with stable cavitation activity at 0.5 MPa, BBB-opening was accompanied by erythrocyte extravasations. In contrast, Group 4 showed that the appearance of BBB-opening and stable cavitation started from 0.2 MPa. However, since the inertial cavitation was not reduced while increasing the acoustic pressure, BBB-opening was produced and accompanied with mild erythrocyte extravasations at 0.3 MPa. Another noteworthy point was that in Group 4, increasing pressure from 1 to 1.5 MPa induced a dramatic increase in inertial cavitation (from 50% to 110%), which caused erythrocyte extravasations to become the dominant effect (Figure 4A and 4B).

Group 1-2 showed a smaller BBB-opening area than Group 3–4 due to the smaller sample volume of the 10-MHz FUS beam compared to the 1-MHz FUS beam (at 1.5 MPa, the width of the BBB-opening area was 1 mm for 10-MHz FUS and 5 mm for 1-MHz FUS). This finding suggested that 10-MHz FUS introduced more local and precise BBB-opening. The change in EB extravasation due to the activity of in-house submicron bubbles was highly correlated with the subharmonic-frequency signal increase but not with ICD. There was almost no change in ICD despite a profound increase in EB extravasation, indicating that inertial cavitation was only weakly correlated with BBB-opening (Figure 3C). In addition, at the BBB-opening threshold of Group 3 and Group 4 (0.3 and 0.2 MPa), there were only subharmonic-frequency signal be detected. Therefore, our data provided convincing evidence that the occurrence of stable cavitation was responsible for BBB-opening, whereas erythrocyte extravasations were mainly caused by inertial cavitation.

### Safety Assessment

**Figure 5:**
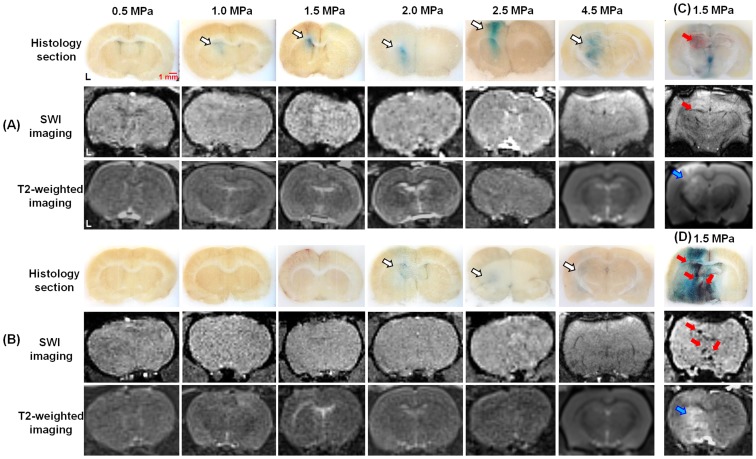
MRI results after FUS sonication. A: In-house submicron bubbles with 10-MHz FUS sonication. B: SonoVue with 10-MHz FUS sonication. C: In-house submicron bubbles with 1-MHz FUS sonication. D: SonoVue with 1-MHz FUS sonication. (white arrows: the BBB-opening region; red arrows: areas of erythrocyte extravasations; blue arrows: brain edema).

We did not detect any alterations in SWI and T2-weighted imaging at the BBB-opening location in Group 1 (Figure 5A), indicating the absence of erythrocyte extravasations and brain anatomical changes at all acoustic pressure levels. In Group 2 (Figure 5B), the mismatch between the FUS frequency and the bubble resonance frequency resulted in BBB-opening occurring at 2.0, 2.5 and 4.5 MPa, and the MRI results therefore did not change with the acoustic pressure level. This finding indicated that 10-MHz FUS was safe because it only produced bubble cavitation activity when the frequencies were matched, and no side effects were observed when the frequencies were mismatched, even at high pressures of up to 4.5 MPa. In contrast, in Group 3, we observed erythrocyte extravasations and brain edema occurrence (using 1.5 MPa as an example) in SWI images (black spots) and T2-weighted images (positive signal changes for edema) respectively. (Figure 5C) In Group 4, severe erythrocyte extravasations and edema were also detected in SWI images and T2-weighted images, individually (Figure 5D).

**Figure 6:**
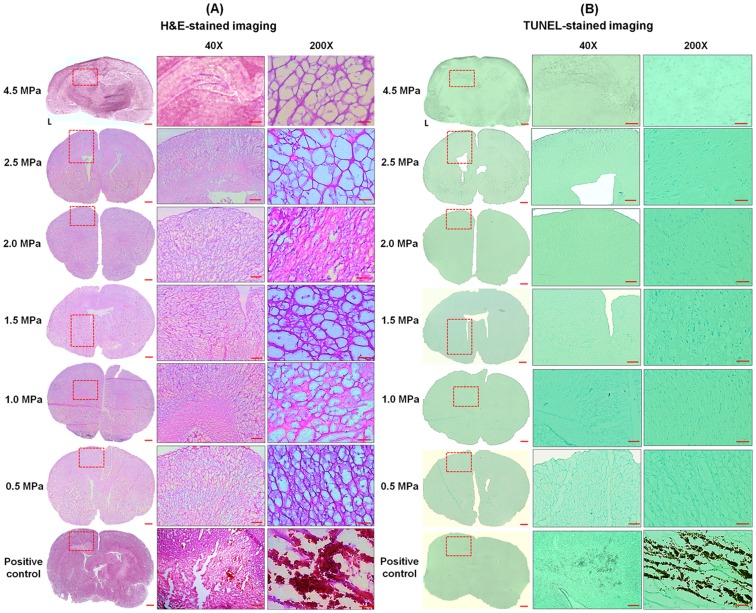
Histological evaluations after BBB-opening. A: H&E-stained and B: TUNEL-stained sections after application of in-house submicron bubbles with 10-MHz FUS sonication. The dotted rectangles indicate the BBB-opening region confirmed by EB extravasation. Positive control: hemorrhage and apoptotic cells (group 4, 1.5 MPa). Scale bars: 1 mm, 1 mm, and 50 µm.

H&E staining and TUNEL assays were used to reveal the extent of erythrocyte extravasations and neuronal apoptosis, respectively. Only mild erythrocyte extravasations were found in Group 1 (Figure 6) at pressures from 2.0 to 4.5 MPa, indicating that the in-house submicron bubbles resonated at 10 MHz indeed did not produce server brain damage or cell apoptosis after FUS sonication.

**Figure 7:**
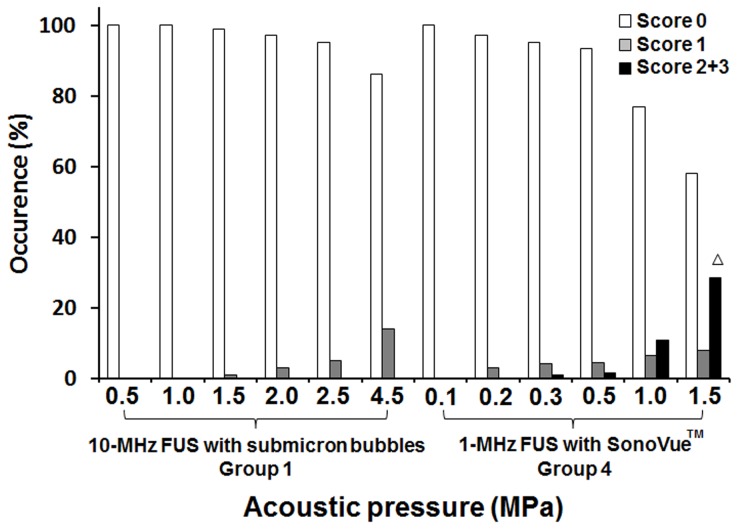
Occurrence of brain hemorrhage according to sonication at different acoustic pressures. Grade 0 indicates no detectable damage, whereas grade 1 denotes erythrocyte extravasations that are considered to be safe. Grades 2 or 3 show hemorrhage regarded as severe. ^Δ^Detected cell apoptosis as assessed by the TUNEL assay.

We then plotted the occurrence and severity of erythrocyte extravasations according to increasing acoustic pressure (Figure 7). In Group 1, the prevalence of grade 0 lesions (intact brain) decreased linearly with increasing acoustic pressure levels. On the other hand, the percentage of mild lesions (grade = 1) increased proportionally to the acoustic pressure level, despite the continued absence of severe hemorrhage (grade = 2 or 3) at the highest applied acoustic pressure (4.5 MPa). In contrast, in Group 4, mild erythrocyte extravasations (grade = 1) could be detected at 0.3 MPa, whereas moderate or severe erythrocyte extravasations (grade = 2 or 3) became apparent with acoustic pressure exceeding 1.0–1.5 MPa. Neuronal apoptosis as detected by TUNEL staining was evident with an acoustic pressure exceeding 1.5 MPa.

### Repetitive and Transcranial FUS Treatment for Enhancing Delivery of Encapsulated Drugs

One possible application of FUS-mediated BBB-opening is the safe and enhanced delivery of drugs such as BCNU to brain tissue. BCNU is a clinically approved chemotherapeutic alkylation agent for treatment of brain tumors, but its efficacy is limited by the inability of delivering a sufficient drug dose to tumors. Its hydrophobic properties allow BCNU to be embedded in the shell of the bubbles and can be attached to the phospholipid shell of bubbles by both electrostatic and hydrophobic interactions without affecting the physical characteristics of the submicron bubbles [22]. BCNU was expected to be locally released by bubble-disruption at a higher pressure level, triggered by 10-MHz FUS exposure. To investigate the feasibility of local drug release by repetitive 10-MHz FUS-triggered bubble oscillations, we therefore synthesized BCNU-loaded submicron-sized bubbles. The safety and efficacy of transcranial and repetitive FUS sonication at the same location was then investigated, with concomitant quantification of drug release by the BCNU-bubbles.

**Figure 8:**
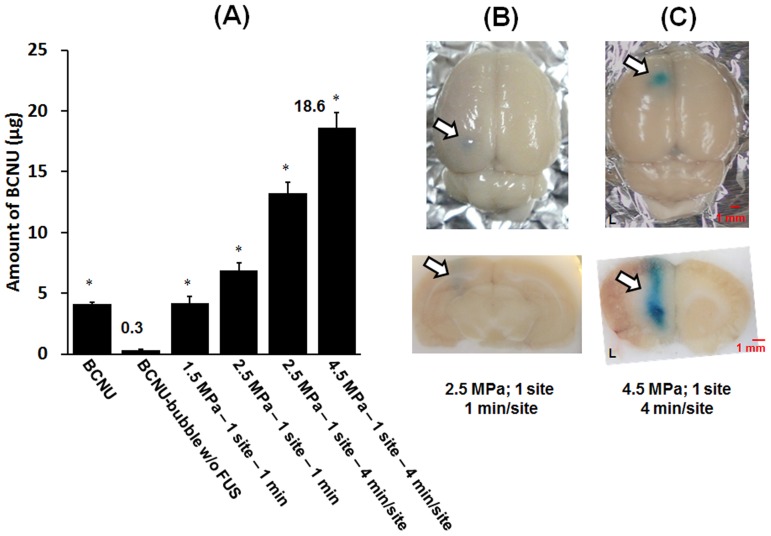
Repetitive and transcranial FUS treatment for enhancing delivery of encapsulated drugs. A: Quantitative evaluation of BCNU release into brain tissue associated with transcranial FUS-triggered destruction of BCNU-bubbles (Single asterisk, *P*<0.05). The corresponding brain sections: B: 2.5 MPa; 1 site; 1 min/site, and C: 4.5 MPa; 1 site; 4 min/site. The treated hemisphere brain was the left brain.

We found that the BCNU encapsulated in bubbles was not released into brain tissues unless its release was triggered by 10-MHz FUS sonication (Figure 8A). The intact BBB of rat brains blocked any noticeable drug accumulation when BCNU was delivered as BCNU-bubble alone (0.31±0.04 µg). Both properties of lipid-soluble and relative small molecular weight (214.1) of BCNU bring it to partially pass through the BBB, depositing in brain tissue (4.61±0.33 µg). Delivery of BCNU to brain tissues could be enhanced by both higher acoustic pressure and a longer time of sonication. Importantly, BCNU release could be improved up to 60 times in comparison to control non-sonicated brains (18.6 µg versus 0.3 µg), and could be enhanced at least 100% comparing to a one-time exposure using the same acoustic pressure. Most notably, the repetitive FUS sonication process significantly enhanced BCNU deposition without causing hemorrhagic damage to the brain. This BBB-opening effect was guaranteed for a total sonication time up to 4 min (Figure 8B and 8C). Thus it is feasible that local drug concentration can be increased without damaging the brain tissue by prolonging FUS irradiation time with drug-loaded bubbles.

## Discussion

The investigation of the bubble activity type (such as stable or inertial cavitation) that leads to different brain effects (such as BBB-opening or erythrocyte extravasations) has been an important issue, since the real use of the FUS-mediated BBB-opening technique for brain drug delivery can be realized only if this mechanism can be fully understood and predictable. It has been shown in several cases that bubbles (2–3 microns in size) can be used to disrupt the BBB at acoustic pressures as low as 0.25–0.3 MPa without evidence of tissue damage or erythrocyte extravasations [31]–[33]. McDannold *et al*. revealed that BBB-opening occurs without inertial cavitation appearance in rabbits [20]. Tung *et al*. discovered that the cavitation activity could be detected in mice and nonhuman primate, and inertial cavitation may not be required for BBB-opening [21], [33]. Wang *et al*. noted that the type and distribution of microbubble might affect the BBB-opening at lower acoustic pressures [34]. Previous reports all pointed out the induction the bubbles on-resonance (or so-called stable cavitation) should play the dominant role of BBB-opening. When further increasing the FUS pressure, erythrocyte extravasations may be accompanied in the BBB-opening process and this should contribute to inertial cavitation. However, a confirmation may not easily be made since two of these bubble activities is usually coupled and mixed in the observation, and is difficult to exclude one from another during the experimental design. One possibility is that the use of about 0.5–1 MHz transducer contains a low inertial cavitation threshold (about 0.066–0.48 MPa) [35], [36]. Also, since the bubbles is nearly impossible to be monodispersed, that magnifies the possibility to have stable and inertial cavitation to be mixed during the FUS exposure. All these causing previous studies have difficulty in making solid and confirmed claims of the playing roles of inertial and stable cavitation.

To characterize the individual role of the inertial and stable cavitation activities, first, we proposed the use of submicron bubbles that fabricated with an improved process to have low size-dispersed distribution to majorly with 350 nm and also the submicron bubbles having the on-resonance frequency close to 10 MHz; second, we employed the 10-MHz FUS so that the pressure threshold of intrinsic inertial cavitation was significantly increased (about 0.62 MPa) [36]. This synergistic approach provided a more reliable evidence to demonstrate that the BBB-opening is dominated by stable cavitation, whereas the erythrocyte extravasation is mainly contributed to inertial cavitation activity.

Our data have demonstrated that the commercial microbubbles with the 1-MHz FUS provided the chance to induce erythrocyte extravasations-accompanied BBB-opening as the pressure was high enough to excite sufficient high inertial cavitation activities (Group 4). On the other hand, exciting FUS with an off-resonant microbubbles did not provide benefits as the pressure required to induce BBB-opening was increased (Groups 2) due to the inefficient induction of bubbles cavitation activities (either stable or inertial cavitation). Of note, the combined use of submicron bubbles and the on-resonance FUS excitation provided merits of selectively triggering stable cavitation (i.e., subharmonics activities) but not inertial cavitation (Group 1; Figure 3B). This is considered to be the optimized FUS-induced BBB-opening conditions since the erythrocyte extravasations can be completely avoid so that the safety can be guaranteed (Figure 3A). On the other hand, the pressure threshold for inertial cavitation of our proposed method is very high, thereby minimizing the risk of inertial cavitation and damage occurring BBB-opening. The wide range of pressures at which BBB-opening can be achieved in the absence of inertial cavitation improves the safety of the procedure.

We also observed that in Group 1 and Group 2, the BBB-opening occurred at pressures of 1.0 and 2.0 MPa, respectively. The reason for this difference of pressure thresholds is not clear. If the major mechanism of BBB-opening was the mechanical stress to the vascular wall caused by steady acoustic-driven bubbles oscillation (stable cavitation), then the matched resonance frequency would be effectively to induce this effect for the submicron bubbles than for the SonoVue bubbles. However, the lowest acoustic pressures inducing BBB-opening in Group 3 and Group 4 were 0.3 and 0.2 MPa, individually. The possible reason for this difference is owing to that the larger bubbles are easier to expand sufficient sizes that could stimulate the vessel walls, and thereby BBB-opening can be achieved at lower acoustic pressures in SonoVue bubbles. In other words, Group 3 showed the erythrocyte extravasations appearance threshold higher than Group 4. The most likely explanation is the smaller size (less than 1 µm) of the in-house submicron bubbles compared to SonoVue bubbles. Bubbles smaller than 2 µm would be likely to fragment at some distance from the endothelial wall when they collapse inside a blood vessel, whereas larger bubbles will expand and fragment while in contact with the endothelial wall. These results pointed out that small size bubbles maybe more safe in low frequency FUS induced BBB-opening process [37], [38].

With submicron bubbles, the BBB-opening threshold of 10-MHz and 1-MHz FUS are 1.0 and 0.3 MPa, respectively. By the contrast, with SonoVue bubbles, the BBB-opening threshold of 10-MHz and 1-MHz FUS are 2.0 and 0.2 MPa, individually. Both two kinds of bubbles show that increasing the FUS frequency of sonication just increases the acoustic pressure required to get BBB-opening. Mcdannold *et al*. found that BBB-opening threshold was the mechanical index (MI) = 0.46 (defined as 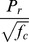, the peak rarefactional pressure (*P*_r_) divided by the square root of the center frequency (*f*_c_)) [39]. Therefore, it is reasonable that increasing sonication FUS frequency would increase BBB-opening threshold. On the other hand, the results of this study also indicated that applying higher frequency FUS increases the safety of BBB-opening due to the MI decrease (Figure 3A; Figure 4A). Thus, the safety of FUS-induced BBB-opening can be improved by manipulating the frequency of FUS.

In Group 2, SonoVue excited at 10-MHz FUS with 4.5 MPa did not reproduce the BBB-opening probably due to the FUS-driving bubble diffusion. Therefore, SonoVue bubbles may be safer than the submicron bubbles since SonoVue bubbles would diffuse before they cause damage. However, the submicron bubbles can produce higher BBB permeability than SonoVue bubbles under all acoustic pressures (Figure 3A). Furthermore, we did not discover the occurrence of brain damage in the submicron bubbles case between 0.1–4.5 MPa (Figure 5A; Figure 6). Taken together, these data suggest that the submicron bubbles still have the potential for effective FUS-induced BBB-opening.

Previous studies have suggested that acoustic-emission signals including harmonics or ultraharmonics could be used to predict the occurrence of BBB-opening [20], [21], [34], [40], [41]. In this study, subharmonic-frequency signals were detected only when BBB-opening occurred. Furthermore, the intensity of the signal was strongly correlated with the quantity of EB extravasation as a function of BBB permeability. The subharmonic content is only produced by bubble oscillations and thus provides a more direct index of stable cavitation. The intensities of these signals might be used to estimate the level of BBB-opening and the location of their occurrence. Therefore, the subharmonic nonlinear component has the potential as a real-time index of BBB-opening.

It was observed that brain regions stained with EB leakage co-localized with the FUS exposure positions (Figure 3, 4, 5, and 6) and with wider distributions than the focal dimension of FUS transducer, which may be possibly due to EB dye leak and diffuse into brain tissue after BBB-opening. Also, the inconsistence of the EB stained positions among animals was due to the small dimension of focal spot induced by the 10-MHz transducer but with a rather wide tissue slicer gap (typically about 1 mm).

The major limitation of this research is the difficulty of applying our results to human brain in current clinical settings, due to the penetration capability of ultrasound frequencies currently in use (i.e., 10-MHz). Indeed the frequency of ultrasound used in this study (10 MHz) is higher than the clinical used 5-MHz ultrasound [42], however, the 7–8 MHz probe has been clinically applied for white matter injury [43] and cerebral palsy [44] diagnostic application, implying the using of 10-MHz therapeutic ultrasound is also possible. On the other hand, the concept of this study may translate to lower resonance frequency of bubble (mono-disperse micron-sized bubbles) and requirement of lower frequency FUS (e.g., 1–5 MHz) which are more relevant for application in human brain. Thereby improve the safety of novel low frequency FUS induced BBB-opening process in the future.

## Conclusions

In this study, the roles of both cavitation activities within FUS-induced BBB-opening process and the feasibility of enhancing local drug release to brain tissue have been confirmed by our designed submicron bubbles and matched the frequency FUS sonication. This study confirmed that inertial cavitation is responsible for brain hemorrhagic damage, and that pure stable cavitation results in BBB-opening. When submicron bubbles are exposed to resonant-frequency matched FUS, inertial cavitation can be reduced, ensuring safe BBB-opening. We also successfully demonstrated that repetitive FUS exposure in combination with drug-loaded submicron bubbles can significantly enhance local chemotherapeutic agent delivery in the brain without the risk of erythrocyte extravasations. These findings are particularly important for increasing the local delivery of high concentrations of drugs to treat CNS diseases, and simultaneously limiting their systemic toxicity. Compared with low frequency FUS (1 MHz), our higher-frequency technique (10 MHz) could provide more precise BBB-opening in brain tissues, suggesting that its potential applications could include local drug delivery in small animals model. This study provides important information towards the goal of successfully translating FUS brain drug delivery into clinical use.

## Supporting Information

File S1 **File includes Method S1, Table S1, and Figures S1-S5.** Table S1: Transducer characteristics and operating frequency ranges. Figure S1: The size distribution of submicron bubble measured by (A) Coulter counter and (B) dynamic light scattering at 4°C or 37°C at different time point (post bubble preparation 0 min, 30 min, 1 h, 2 h and 4 h). Figure S2: The concentration of submicron bubble measured by Coulter counter and at 4°C or 37°C at different time point (post bubble preparation 0 min, 30 min, 1 h, 2 h and 4 h). Figure S3: The experimental setup for measuring the acoustic attenuation of the submicron bubbles. Figure S4: The Attenuation measurement results of submicron bubbles. Figure S5: The acoustic pressure maps of (A) 10-MHz, (B) 5-MHz and (C) 1-MHz transducers, respectively.
(DOCX)
