# Supplementary material for: Submicron-Bubble-Enhanced Focused Ultrasound for Blood–Brain Barrier Disruption and Improved CNS Drug Delivery
Source: PLoS One. 2014 May 2;9(5):e96327. doi: 10.1371/journal.pone.0096327 (PMC4008627; doi:10.1371/journal.pone.0096327)
Supplement: File S1 — File includes Method S1, Table S1, and Figures S1-S5. Table S1: Transducer characteristics and operating frequency ranges. Figure S1: The size distribution of submicron bubble measured by (A) Coulter counter and (B) dynamic light scattering at 4°C or 37°C at different time point (post bubble preparation 0 min, 30 min, 1 h, 2 h and 4 h). Figure S2: The concentration of submicron bubble measured by Coulter counter and at 4°C or 37°C at different time point (post bubble preparation 0 min, 30 min, 1 h, 2 h and 4 h). Figure S3: The experimental setup for measuring the acoustic attenuation of the submicron bubbles. Figure S4: The Attenuation measurement results of submicron bubbles. Figure S5: The acoustic pressure maps of (A) 10-MHz, (B) 5-MHz and (C) 1-MHz transducers, respectively. (DOCX) [file pone.0096327.s001.docx]

**Supplementary Information Method**

**Method S1: Estimation of Submicron Bubbles Resonance Frequency**

The resonance frequency of submicron bubbles was estimated by acoustic attenuation method, an approach described in detail elsewhere^1-2^. It has been shown that higher spectral attenuation of an acoustic pulse can be measured around the resonance frequencies of bubbles. The five commercially available focused ultrasound (FUS) transducers (Panametrics Inc., Waltham, MA, USA) were used to cover the frequency bandwidth (-6 dB) ranging from 2.2 to 41.6 MHz (Table S1). An overview of the hardware configuration is shown in Figure S3. An arbitrary waveform generator (AWG2041, Tektronix, Beaverton, OR, USA) was used to generate pulses, which were amplified with an RF power amplifier (325LA, Electronics & Innovation, Rochester, NY, USA) and then exciting transducers. Submicron bubbles were situated in a sample chamber located within the FUS beam, which entered the chamber through acoustically transparent polyurethane membrane window (on both sides of the chamber). Acoustic pulses traveling through the chamber were received on the opposite side were acquired by a hydrophone (MHA9-150, Force, Demmark) and then digitized with an oscilloscope (LT322, LeCory Corporation, Chestnut Ridge, NY, USA). The attenuation spectrum was obtained by comparing the Fourier transforms of received signals in absence and presence of bubbles. Only the results at frequencies within the –6-dB bandwidth of each transducer were adopted to ensure the reliability of the measurement. These data were stored in the personal computer for off-line processing by the MATLAB software (Mathworks, Natick, MA). The pulses were with a 50-cycle length, a pulse repetition frequency of 50 Hz, and an acoustic pressure of 50 kPa. The concentration of submicron bubbles was 0.0005 v/v%. The attenuation coefficient (α_mean_) of submicron bubbles was calculated by following formula:

$\alpha_{\mathrm{mean}}(f)=-\frac{20}{L}Log(\frac{P_{S\mathrm{Bs}}}{P_{\mathrm{water}}})$ (S.1)

where L is the length of sample chamber; P_SBs_ is the acoustic pressure of signal attenuated by submicron bubbles; and P_water_ is the acoustic pressure of signal attenuated by water.

The results are shown in Figure S4. The attenuation coefficients decreased with frequency increasing from 6 to 40 MHz due to the in-house submicron bubbles is not mono-dispersed. The peak values (-6 dB) of attenuation coefficients are at the vicinity of 6 to 12 MHz. Therefore, the resonance frequency of submicron bubbles was about 10 MHz.

**Supplementary Information Tables and Figures**

**Table S1.** Transducer characteristics and operating frequency ranges

| Transducer model | Central frequency (MHz) | Focal length  (mm) | Aperture (mm) | Bandwidth  (-6 dB, MHz) |
| --- | --- | --- | --- | --- |
| V310 | 5 | 20.5 | 6.4 | 2.2 – 6.8 |
| V321 | 7.5 | 50 | 19 | 5.0 – 10.0 |
| V322 | 10 | 50 | 20.5 | 6.5 – 13.2 |
| V319 | 15 | 50 | 12.7 | 10.5 – 19.4 |
| V375 | 30 | 19 | 6.4 | 18.0 – 41.6 |


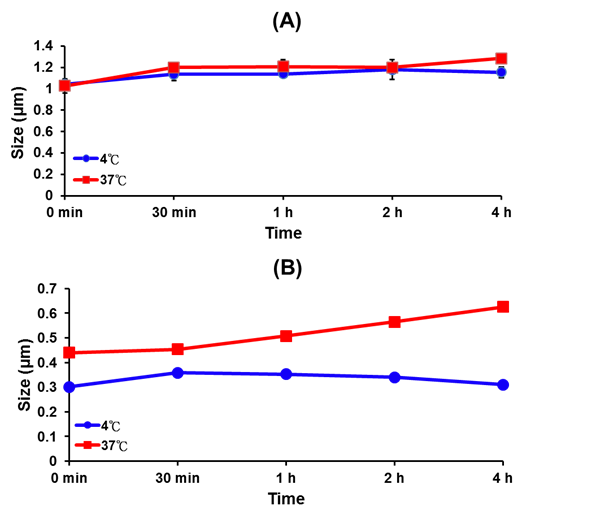


**Figure S1.** The size distribution of submicron bubble measured by (A) Coulter counter and (B) dynamic light scattering at 4 °C or 37 °C at different time point (post bubble preparation 0 min, 30 min, 1 h, 2 h and 4 h).


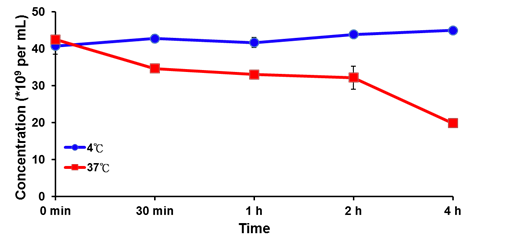


**Figure S2.** The concentration of submicron bubble measured by Coulter counter and at 4 °C or 37 °C at different time point (post bubble preparation 0 min, 30 min, 1 h, 2 h and 4 h).


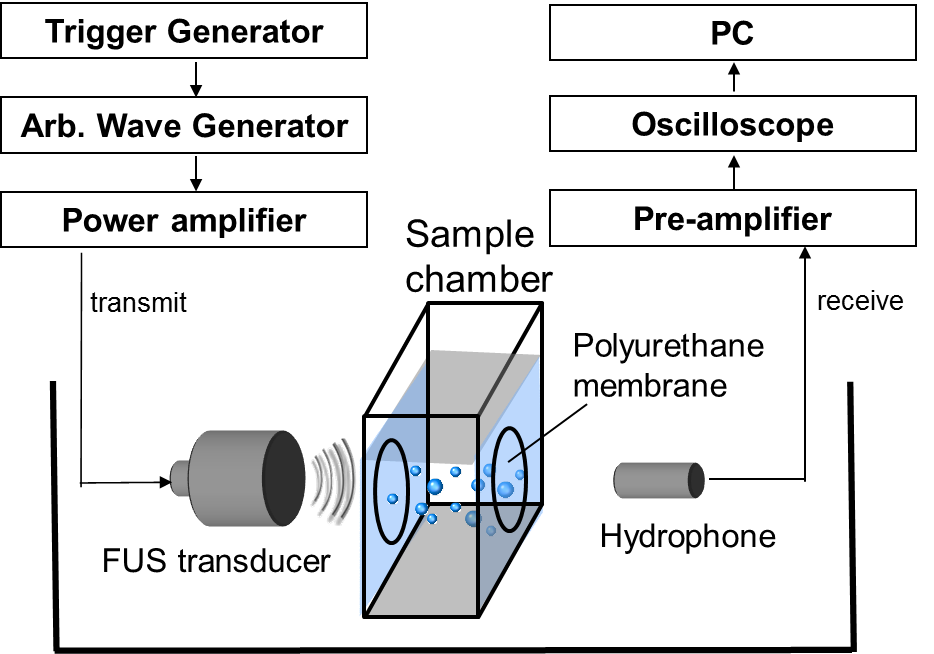


**Figure S3.** The experimental setup for measuring the acoustic attenuation of the submicron bubbles.


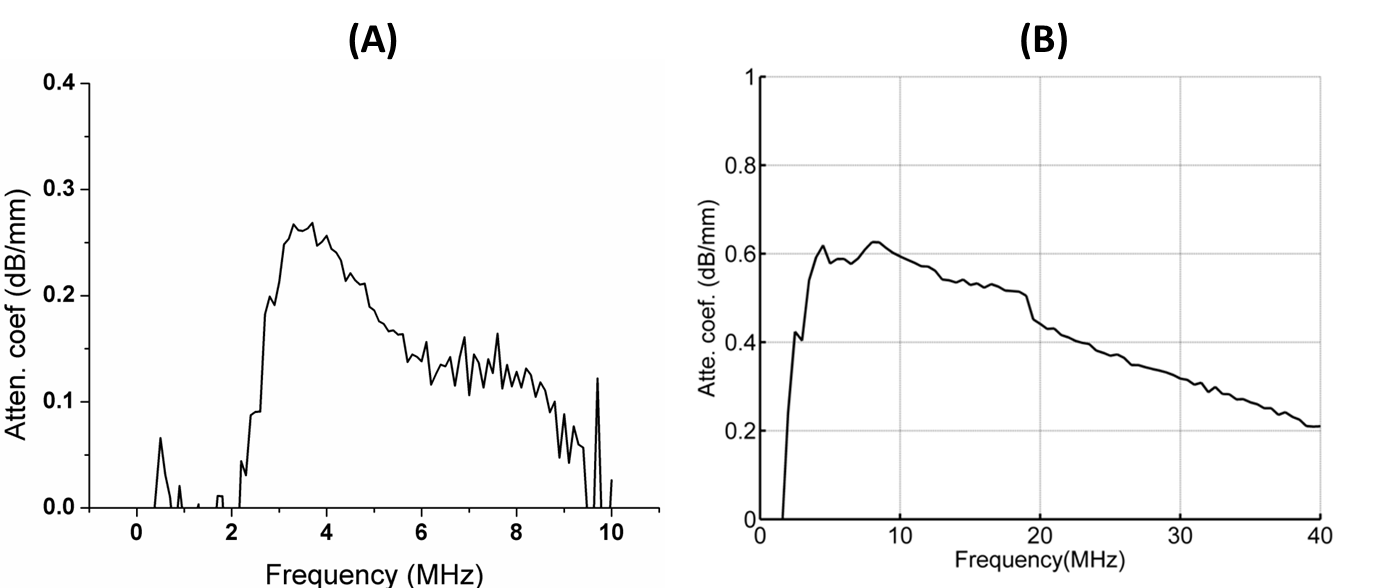


**Figure S4.** The Attenuation measurement results of submicron bubbles.


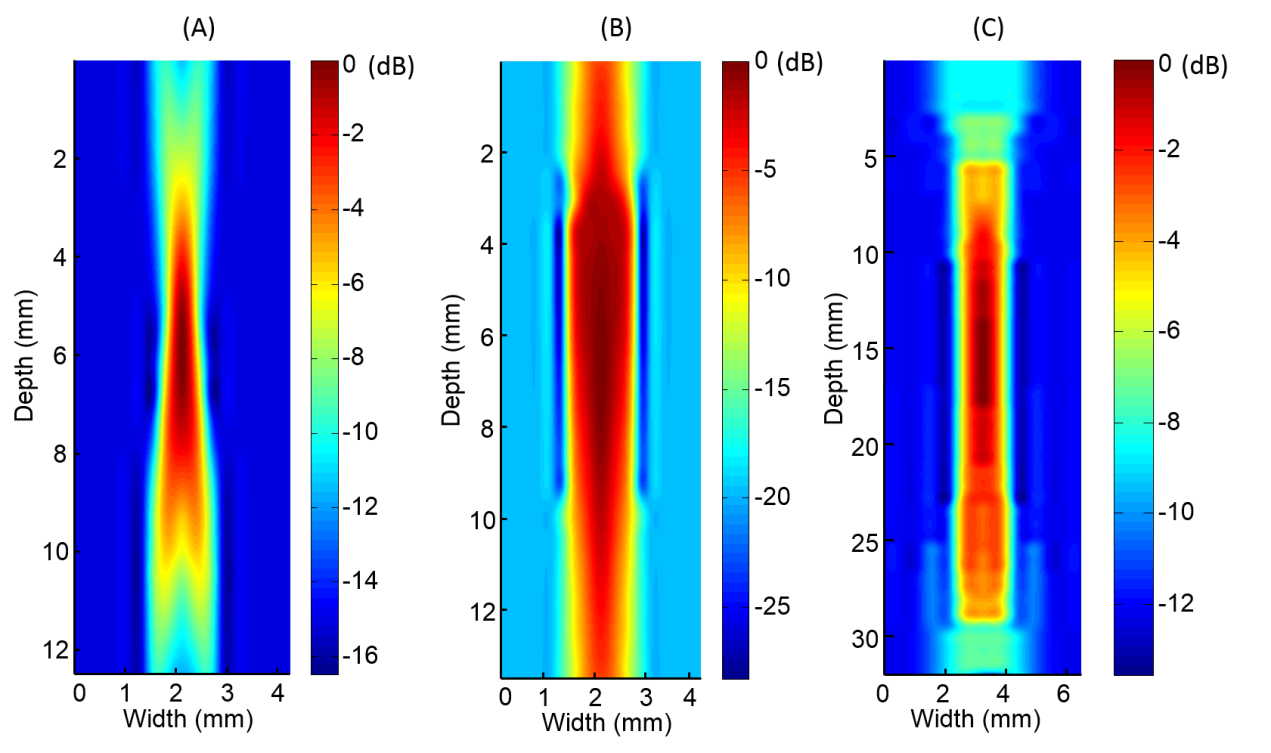


**Figure S5**. The acoustic pressure maps of (A) 10-MHz, (B) 5-MHz and (C) 1-MHz transducers, respectively.

[1]de Jong, N.; Hoff, L.; Skotland, T.; Bom, N.; Absorption and scatter from microspheres: Theoretical considerations and some measurements. *Ultrasonics*. **1992**, 30, 95-103.

[2] Goertz ,D.E.; de Jong, N.; van der Steen, A.F.; Attenuation and size distribution measurements of Definity^TM^ and manipulated Definity^TM^ populations, [*Ultrasound Med Biol.*](javascript:AL_get(this,%20'jour',%20'Ultrasound%20Med%20Biol.');) **2007**, 33, 1376-1388.
